# Short-chain fatty acids and inulin, but not guar gum, prevent diet-induced obesity and insulin resistance through differential mechanisms in mice

**DOI:** 10.1038/s41598-017-06447-x

**Published:** 2017-07-21

**Authors:** Karolin Weitkunat, Christin Stuhlmann, Anna Postel, Sandra Rumberger, Maria Fankhänel, Anni Woting, Klaus Jürgen Petzke, Sabrina Gohlke, Tim J. Schulz, Michael Blaut, Susanne Klaus, Sara Schumann

**Affiliations:** 1 Department Physiology of Energy Metabolism, German Institute of Human Nutrition in Potsdam Rehbruecke, Arthur-Scheunert-Allee 114-116, 14558 Nuthetal, Germany; 2 Department Gastrointestinal Microbiology, German Institute of Human Nutrition in Potsdam Rehbruecke, Arthur-Scheunert-Allee 114-116, 14558 Nuthetal, Germany; 3 Department of Adipocyte Development and Nutrition, German Institute of Human Nutrition in Potsdam Rehbruecke, Arthur-Scheunert-Allee 114-116, 14558 Nuthetal, Germany

## Abstract

The role of dietary fibre and short-chain fatty acids (SCFA) in obesity development is controversially discussed. Here, we investigated how various types of dietary fibre and different SCFA ratios affect metabolic syndrome-related disorders. Male mice (B6) were fed high-fat diets supplemented with dietary fibres (either cellulose, inulin or guar gum) or different Ac:Pr ratios (high acetate (HAc) or propionate (HPr)) for 30 weeks. Body-fat gain and insulin resistance were greatly reduced by inulin, but not by guar gum, and completely prevented by SCFA supplementation. Only inulin and HAc increased body temperature, possibly by the induction of beige/browning markers in WAT. In addition, inulin and SCFA lowered hepatic triglycerides and improved insulin sensitivity. Both, inulin and HAc reduced hepatic fatty acid uptake, while only inulin enhanced mitochondrial capacity and only HAc suppressed lipogenesis in liver. Interestingly, HPr was accompanied by the induction of Nrg4 in BAT. Fermentable fibre supplementation increased the abundance of bifidobacteria; *B*. *animalis* was particularly stimulated by inulin and *B*. *pseudolongum* by guar gum. We conclude that in contrast to guar gum, inulin and SCFA prevent the onset of diet-induced weight gain and hepatic steatosis by different mechanisms on liver and adipose tissue metabolism.

## Introduction

Low dietary fibre consumption in Western countries is accompanied by an increased prevalence of obesity and insulin resistance^1, 2^. Hence, high dietary fibre intake is associated with a lower body weight in humans, which is supposed to be the result of a reduced appetite^3, 4^. However, a systematic review revealed that dietary fibre intake enhanced satiety in only 39% of all published studies^5^. Thus, besides the positive effects on gastric filling and intestinal motility, additional metabolic effects have to exist. One postulated mechanism relates to the production of short-chain fatty acids (SCFA), mainly acetate (Ac) and propionate (Pr). These SCFA are formed from dietary fibre as a result of intestinal fermentation. They affect a variety of biological processes in multiple organs/tissues. While propionate is primarily a precursor for (intestinal) gluconeogenesis^6, 7^ and an inhibitor of hepatic lipid synthesis^8^, acetate was also shown to increase energy expenditure by activating AMP-activated protein kinase (AMPK) in liver and muscle^9, 10^. Furthermore, recent literature data claim that acetate increases the activity of brown fat and induces the formation of “beige adipocytes”^11–13^.

Dietary fibres include a wide variety of substances, in particular complex carbohydrates. This may explains why the effects of different fibre types on diet-induced obesity appear contradictory. On the one hand it was shown that supplementation of guar gum results in an obese phenotype^14^, while on the other hand it was demonstrated that guar gum protects against high-fat (HF) diet-induced obesity^15^. A study using PolyGlycopleX showed no effect on obesity development^16^, while supplementation of bamboo shoot fibre^17^, beta-glucan, fructo-oligosaccharide, or pectin^18^ protected against HF-induced weight gain. Previously we found that supplementation of inulin (fermentable dietary fibre) reduced the hepatic expression of genes involved in lipogenesis and fatty acid elongation/desaturation, while cellulose (non-fermentable fibre) showed no effects^19^. Although there were no effects on body weight/fat, inulin supplementation increased SCFA formation and reduced the Ac:Pr ratio in caecal contents and portal vein plasma. Based on these results we hypothesised that prolonged inulin supplementation might repress the onset of diet-induced obesity and related disorders, such as hepatic steatosis and insulin resistance. Furthermore we supposed that the Ac:Pr ratio is important for the observed effects. We examined this in C3H mice by feeding HF diets that were supplemented with either a high Ac or a high Pr ratio for 6 and 22 weeks (wks). The results showed that especially a high Pr concentration reduced HF-induced hepatic steatosis and insulin resistance^20^.

Hence, we assume that depending on the fibre type, various total SCFA amounts and different Ac:Pr ratios are formed which in turn could be responsible for the controversial literature data regarding dietary fibre effects on obesity. Following this hypothesis, we investigated in the present study whether different types of dietary fibre and different ratios of Ac:Pr affect the development of obesity in the long term. Besides non-fermentable cellulose, we used two fermentable fibres: inulin (composed mainly of fructose units) exhibiting low viscosity and guar gum (composed of galactose and mannose units) exhibiting high viscosity. Male mice (C57BL/6JRj) were fed semi-synthetic HF diets supplemented either with cellulose (HFC), inulin (HFI) or guar gum (HFG) for 30 wks. To clarify whether the Ac:Pr ratio is of importance, we additionally included mice that were supplemented with a high acetate ratio (HAc; 10:1 Ac:Pr) or a high propionate ratio (HPr; 1:2.5 Ac:Pr). We investigated the effects on body weight/fat as well as glucose homeostasis and analysed liver and adipose tissue metabolism. Furthermore, we examined the intestinal microbiota composition in faecal samples.

## Results

### Inulin and SCFA attenuate high-fat diet-induced body weight/fat gain and improve insulin resistance

**Figure 1:**
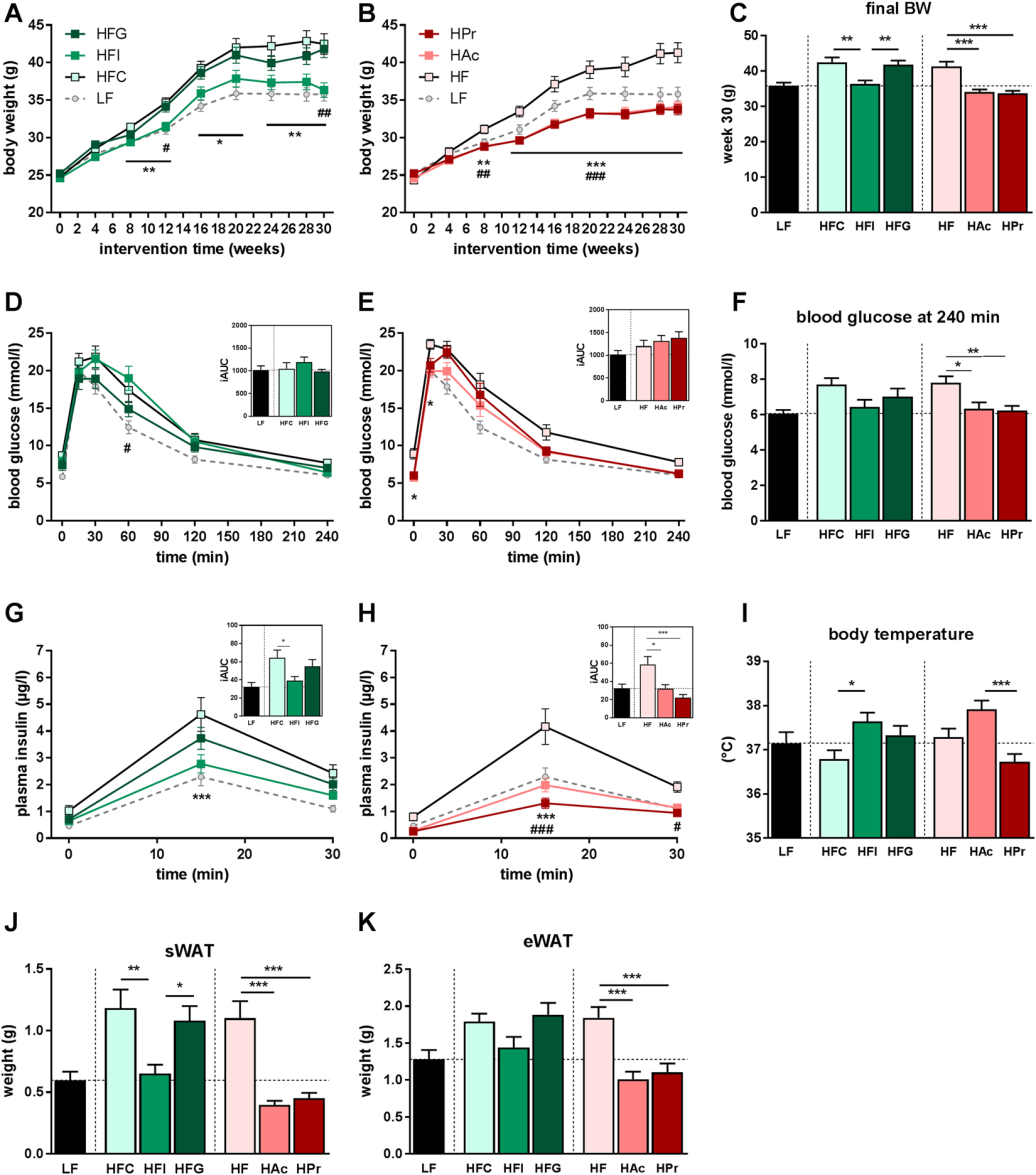
Inulin and SCFA prevent diet-induced body weight/fat gain and insulin resistance. Male C57BL/6JRj mice were fed a semi-synthetic low-fat diet (LF) or high-fat diets (HF) supplemented with either 10% dietary fibre (HFC: 10% cellulose; HFI: 3% cellulose + 7% inulin; HFG: 3% cellulose + 7% guar gum; depicted in green hues) or 5% SCFA (depicted in red hues) with different Ac:Pr ratios, a high acetate (HAc; 10:1 Ac:Pr) or high propionate diet (HPr; 1:2.5 Ac:Pr). Body weight development during (**A**) dietary fibre, (**B**) SCFA intervention or (**C**) final body weight (BW) after 30 weeks, n = 20–22. (**D**–**F**) Time-dependent blood glucose levels and (**G**, **H**) insulin concentrations after oral glucose load (2 g/kg body weight) and the corresponding incremental area under the curves (iAUC), n = 10–11. (**I**) Rectal body temperature was measured in week 29 of intervention, n = 19–21. Tissue weights of (**J**) subcutaneous white adipose tissue (sWAT) and (**K**) epididymal WAT (eWAT) adipose tissue (BAT) after 30 weeks, n = 19–21. Data are mean +/− SEM, */^#^P < 0.05; **/^##^P < 0.01, ***/^###^P < 0.001. *Significant differences HFC vs. HFI or HF vs. HAc, ^#^significant differences HFI vs. HFG or HF vs. HPr.

At first we examined whether inulin or guar gum affect HF diet-induced obesity and associated disorders. From wk 8 on, HFC feeding led to an increased body weight compared to LF mice (Fig. 1A,C). Inulin, but not guar gum supplementation reduced the body weight to LF level. SCFA supplementation resulted in a reduced body-weight gain and final body weight compared to the HF-group no matter whether a high Ac or a high Pr ratio was applied (Fig. 1B,C). Although cumulative feed intake as well as energy intake were slightly reduced in all experimental feeding groups, these changes are unlikely to account for the striking body weight differences (Table 1). Diet digestibility of the diets supplemented with inulin or guar gum were higher than that of the HFC diet. In contrast, SCFA supplementation did not affect diet digestibility. Energy expenditure (measured in wk 10) was not significantly different among the groups and the changes in respiratory quotient (RQ), which were observed only at night, are reflecting the changes in diet composition. To evaluate the effects of dietary fibre and SCFA on glucose homeostasis, an oral glucose tolerance test (OGTT) was performed in wk 20 of intervention. Homeostatic model assessment value for insulin resistance (HOMA-IR) was calculated from the basal levels, indicating an improved insulin sensitivity in both SCFA and in tendency also in the dietary fibre groups (Table 1). While the incremental area under the curve (iAUC) of blood glucose during the OGTT was not affected (Fig. 1D,E), inulin (in tendency) as well as Ac and Pr supplementation reduced blood glucose concentrations 240 min after gavage (Fig. 1F). Furthermore, HFI as well as HAc and HPr mice had reduced plasma insulin levels after 15 min (Fig. 1G,H). Hence, inulin and SCFA supplementation increased the efficiency of glucose clearance while requiring lower insulin levels, indicating that the insulin sensitivity in these groups may have improved. Interestingly, rectal body temperature (measured in wk 29) was increased in HFI and HAc mice, while guar gum and HPr had no effect (Fig. 1I). Plasma parameters after 30 wks of intervention were not improved by dietary fibre or SCFA supplementation (Table 1). In accordance with total body fat data (Table 1), the HF diet-induced increase of subcutaneous and epididymal white adipose tissue (sWAT, eWAT) weight was attenuated by inulin and completely prevented by Ac/Pr, but not by guar gum supplementation (Fig. 1J,K).

**Table 1:** Inulin and SCFA diminish fat mass gain, while only propionate reduces the RQ level during night.

	LF	HFC	HFI	HFG	P-value^1^	HF	HAc	HPr	P-value^2^
Body fat mass (g)	6.46 ± 0.90	11.68 ± 1.27^a^	6.62 ± 0.79^b^	10.57 ± 1.06^a^	<0.01	10.76 ± 1.21^a^	4.27 ± 0.51^b^	4.64 ± 0.60^b^	<0.001
Lean body mass (g)	29.36 ± 0.41	31.04 ± 0.28	29.99 ± 0.33	31.26 ± 0.37	ns	30.57 ± 0.56	29.85 ± 0.31	29.11 ± 0.48	ns
Cum feed intake (g/3d)	8.23 ± 0.30	7.11 ± 0.17	6.49 ± 0.35	6.39 ± 0.40	ns	6.95 ± 0.25	7.07 ± 0.45	6.22 ± 0.29	ns
Energy intake (kJ/d)	47.4 ± 2.3	52.7 ± 2.0	49.8 ± 2.8	44.6 ± 4.1	ns	46.7 ± 2.9	44.1 ± 5.4	42.2 ± 3.3	ns
EE Day (kJ/h/kg lean mass)	51.8 ± 1.4	55.5 ± 1.3	56.2 ± 1.8	53.0 ± 1.4	ns	51.4 ± 1.6	51.6 ± 1.7	51.4 ± 1.1	ns
EE Night (kJ/h/kg lean mass)	69.2 ± 2.1	67.9 ± 1.5	70.5 ± 1.5	66.7 ± 1.9	ns	66.4 ± 2.4	66.3 ± 0.8	66.1 ± 1.7	ns
RQ Day	0.88 ± 0.02	0.84 ± 0.01	0.83 ± 0.01	0.85 ± 0.01	ns	0.83 ± 0.01	0.82 ± 0.01	0.81 ± 0.01	ns
RQ Night	1.00 ± 0.01	0.86 ± 0.00^a^	0.89 ± 0.01^b^	0.88 ± 0.01^a,b^	<0.05	0.87 ± 0.00^a^	0.85 ± 0.01^b^	0.83 ± 0.01^b^	<0.05
Faeces excretion (g/d)	0.11 ± 0.01	0.26 ± 0.04	0.22 ± 0.02	0.19 ± 0.01	ns	0.30 ± 0.027^a^	0.23 ± 0.01^b^	0.26 ± 0.02^a,b^	<0.05
Faeces energy content (kJ/g)	15.55 ± 0.26	16.03 ± 0.12	16.90 ± 0.31	15.93 ± 0.52	ns	13.49 ± 0.17	13.40 ± 0.07	13.55 ± 0.17	ns
Diet digestibility (%)	92.7 ± 0.6	90.6 ± 0.2^a^	93.4 ± 0.6^b^	94.6 ± 0.4^b^	<0.05	94.5 ± 0.2	94.4 ± 0.1	94.2 ± 0.3	ns
Plasma triglycerides (mmol/l)	0.19 ± 0.03	0.15 ± 0.03	0.13 ± 0.02	0.14 ± 0.03	ns	0.14 ± 0.03	0.21 ± 0.02	0.16 ± 0.03	ns
Plasma cholesterol (mg/dl)	87.8 ± 6.8	117.1 ± 12.8^a^	81.0 ± 5.4^b^	108.2 ± 6.5^a,b^	<0.05	131.6 ± 8.0	115.0 ± 7.6	118.9 ± 7.3	ns
Plasma free fatty acids (mmol/l)	0.83 ± 0.06	0.66 ± 0.03	0.69 ± 0.04	0.58 ± 0.03	ns	0.74 ± 0.04	0.90 ± 0.06	0.75 ± 0.08	ns
HOMA-IR	2.85 ± 0.48	10,18 ± 2.23	5.91 ± 1.39	5.75 ± 0.95	ns	8.32 ± 1.97	1.85 ± 0.50	1.61 ± 0.41	<0.05

Table shows the summary of body composition (wk 30; n = 20–22), energy homeostasis (wk 10; n = 10–11) and plasma parameters (wk 30; n = 10), as well as HOMA-IR values (wk 20; n = 10–11) of male C57BL/6JRj mice fed a semi-synthetic low-fat diet (LF) or high-fat diets (HF) supplemented with either 10% dietary fibre (HFC: 10% cellulose; HFI: 3% cellulose + 7% inulin; HFG: 3% cellulose + 7% guar gum) or 5% SCFA with different Ac:Pr ratios, a high acetate (HAc; 10:1 Ac:Pr) or high propionate diet (HPr; 1:2.5 Ac:Pr) for 30 weeks. Data are mean +/− SEM. Statistical tests were performed among ^1^dietary fibre groups (HFC, HFI, HFG) and ^2^SCFA-groups (HF, HAc, HPr); Cum: cumulative; EE: energy expenditure; RQ: respiratory quotient; HOMA-IR: homeostatic model assessment value for insulin resistance.

### Inulin and SCFA affect adipose tissue function

**Figure 2:**
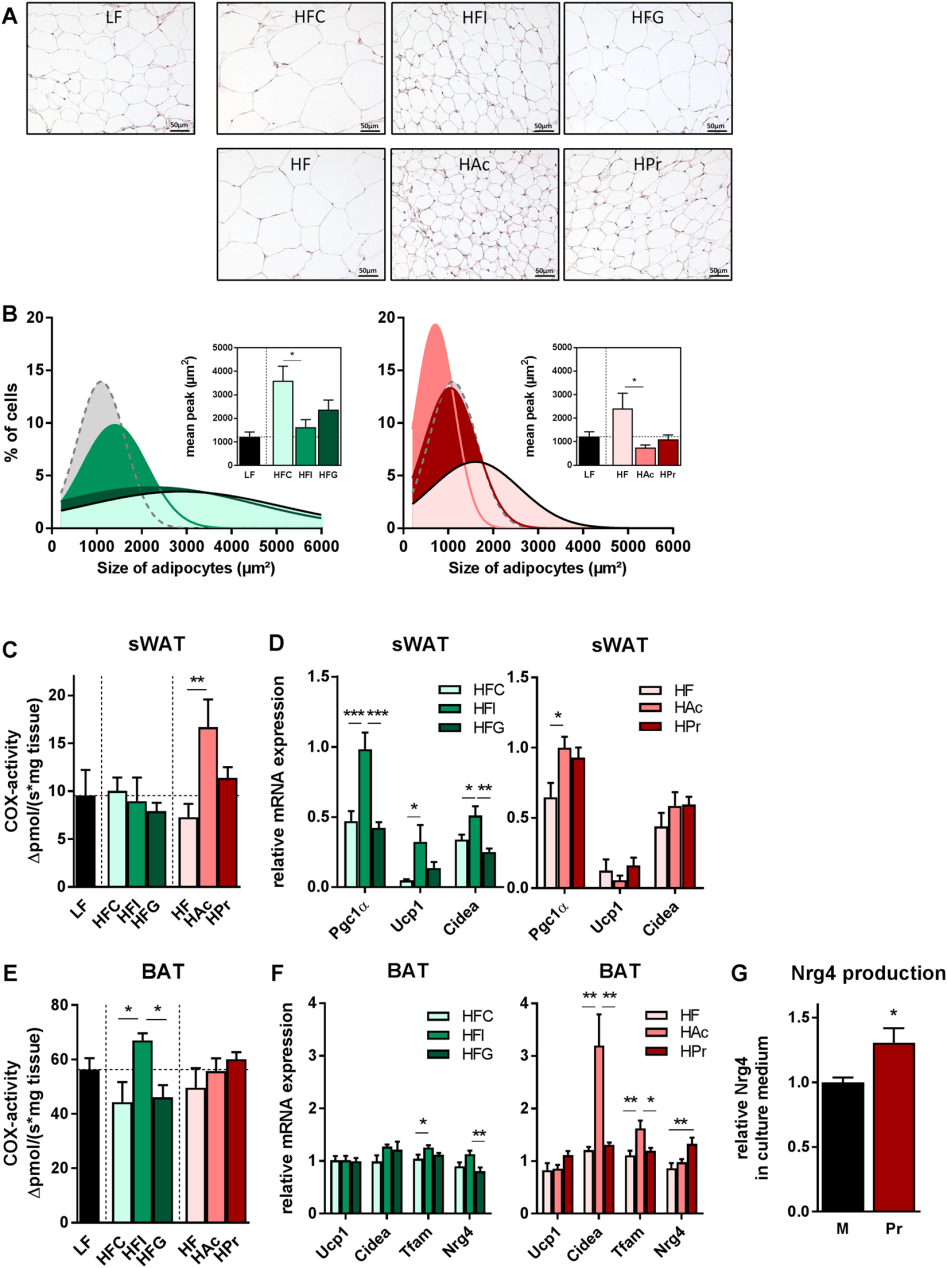
Inulin and SCFA reduce adipocyte size and affect adipose tissues metabolism by distinct mechanisms. Characterization of (**A**–**D**) subcutaneous white adipose tissue (sWAT) and (**E**,**F**) brown adipose tissue (BAT) of male C57BL/6JRj mice that were fed a semi-synthetic low-fat diet (LF) or high-fat diets (HF) supplemented with either 10% dietary fibre (HFC: 10% cellulose; HFI: 3% cellulose + 7% inulin; HFG: 3% cellulose + 7% guar gum; depicted in green hues) or 5% SCFA (depicted in red hues) with different Ac:Pr ratios, a high acetate (HAc; 10:1 Ac:Pr) or high propionate diet (HPr; 1:2.5 Ac:Pr) for 30 weeks. (**A**) Representative histological images of sWAT. (**B**) Adipocyte size distribution and corresponding quantification of cell size, n = 5. Measurement of cytochrome c oxidase (COX) activity in (**C**) sWAT and (**E**) BAT, n = 5. Analysis of mRNA expression in (**D**) sWAT or (**F**) BAT, normalized to LF-diet (set as 1), n = 8–9. (**G**) Relative Nrg4 secretion of primary brown adipocytes after 4 h of treatment with propionate (Pr) or without treatment (M, Media), n = 5. Data are mean + SEM, *P < 0.05; **P < 0.01; ***P < 0.001.

To elucidate the underlying mechanisms leading to the observed phenotype, we first concentrated on the adipose tissue. The elevated body temperature in the HFI and HAc group indicates an increased brown adipose tissue (BAT) activity and/or “browning” of the white adipose tissue which is usually most prominent in the subcutaneous depot^21^. Histological examination of sWAT suggested a reduced adipocyte hyperplasia in HFI and both SCFA groups (Fig. 2A). Determination of adipocyte size revealed a higher abundance of small adipocytes and correspondingly a lower abundance of large adipocytes in HAc- and inulin-fed mice (Fig. 2B). Guar gum showed no effect on adipocyte hyperplasia. For closer scrutiny of possible “browning” we investigated mitochondrial respiratory capacity by measuring cytochrome c oxidase (COX) activity (Fig. 2C) as well as gene expression of “browning” markers (Pgc1α, Ucp1, Cidea; Fig. 2D). Inulin had no effect on COX activity but increased gene expression of all three “browning” markers. In the HAc group, COX activity as well as Pgc1α expression was elevated, while guar gum and HPr showed no effect at all.

Indicating a high ectopic lipid accumulation, BAT weight was increased in HFC, HFG and HF animals, while inulin and SCFA supplementation led to a BAT weight that was comparable to the LFD group (Supplementary Figure 1). This effect was previously described as “whitening” which is accompanied by a decreased expression of vascular endothelial growth factor A (Vegfα)^22^. Indeed, we determined a reduced Vegfα gene expression in the HFC, HFG and HF group. Furthermore, leptin gene expression as marker for an increased presence of white adipocytes within the BAT was elevated in the high-fat control groups. To examine BAT activity in more detail, COX activity and several gene expression markers were determined. Whereas COX activity was increased after inulin supplementation (Fig. 2E), UCP1 gene expression was not affected by either treatment. HAc feeding led to a strong increase in Cidea expression and additionally elevated the mitochondrial marker, Tfam, similar to inulin (Fig. 2F). Additionally, we investigated the role of the recently discovered batokine, neuregulin 4 (Nrg4), which was shown to be associated with the development of diet-induced obesity in humans and mice^23, 24^. Nrg4 gene expression was induced in HFI and HPr fed mice. BAT Nrg4 gene expression further correlated with odd-chain fatty acid (OCFA) levels, indicating a connection between Pr formation and Nrg4 (Supplementary Figure 2A). Using primary brown adipocytes, we investigated this connection in more detail and found that incubation with 1 mM Pr led to a 1.3-fold increase in BAT-derived Nrg4 secretion after 4 h (Fig. 2G).

### Inulin and SCFA alter the hepatic lipid metabolism

**Figure 3:**
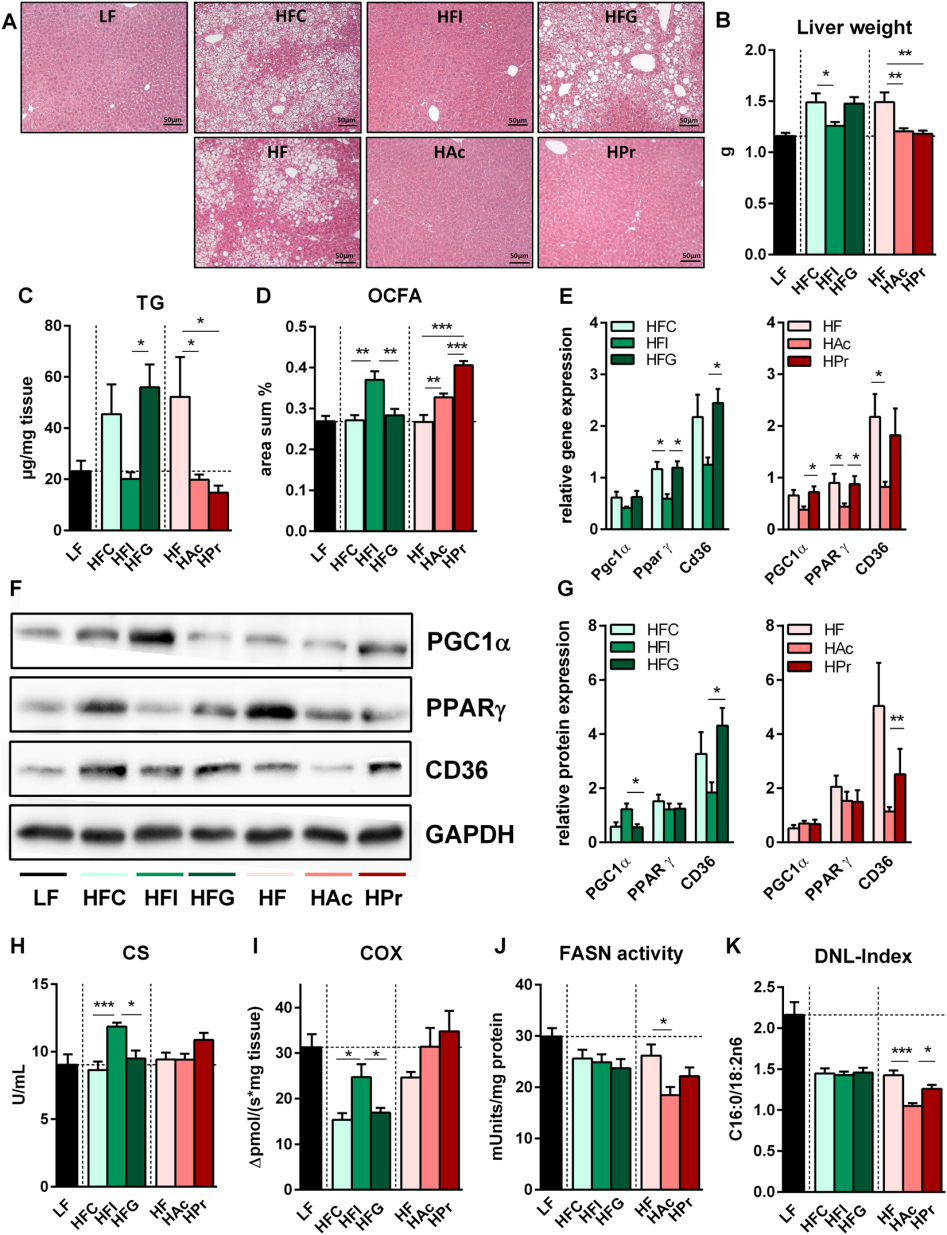
Inulin and SCFA prevent the onset of diet-induced hepatic steatosis by affecting lipid metabolism in different ways. Characterization of liver tissue in male C57BL/6JRj mice that were fed a semi-synthetic low-fat diet (LF) or high-fat diets (HF) supplemented with either 10% dietary fibre (HFC: 10% cellulose; HFI: 3% cellulose + 7% inulin; HFG: 3% cellulose + 7% guar gum; depicted in green hues) or 5% SCFA (depicted in red hues) with different Ac:Pr ratios, a high acetate (HAc; 10:1 Ac:Pr) or high propionate diet (HPr; 1:2.5 Ac:Pr) for 30 weeks. (**A**) Representative H&E staining of hepatocytes after intervention. (**B**) Liver tissue weight (n = 19–21) and corresponding (**C**) hepatic triglyceride (TG) concentration, n = 9–11. (**D**) Formation of odd-chain fatty acids (OCFA) in liver phospholipid fraction, n = 8. (**E**) Hepatic gene expression, (**F**) respective western blots with corresponding load control (GAPDH) and (**G**) analysis of protein expression (normalized to LF-diet as set to a value of 1), n = 4–8. Analysis of (**H**) citrate synthase (CS) and (**I**) cytochrome c oxidase (COX) activity in liver, n = 5–8. (**J**) Hepatic fatty acid synthase (FASN) activity and (**K**) calculated de novo lipogenesis (DNL)-Index by measurement of long-chain fatty acid amount in liver phospholipids, n = 8. Data are mean + SEM. *P < 0.05; **P < 0.01; ***P < 0.001.

Since we previously observed beneficial effects of inulin and SCFA supplementation on HF-induced hepatic steatosis^19, 20^, we investigated the liver metabolism in more detail. While guar gum supplementation showed no effect, inulin as well as SCFA feeding reduced total liver weight due to decreased hepatic fat accumulation (Fig. 3A–C). Odd-chain fatty acid (OCFA) levels as dose-dependent marker for hepatic Pr metabolism^25^ were increased with higher dietary Pr concentrations in HAc, HPr and HFI mice (Fig. 3D). Guar gum feeding did not increase OCFA levels, indicating that hepatic propionate availability was not increased, possibly due to a reduced intestinal Pr formation by intestinal fermentation.

We next examined whether the observed effects were due to differences in hepatic lipid metabolism. As indicated by a higher Pgc1α protein, but not gene expression (Fig. 3E–G) and an increased citrate synthase (CS)/COX activity (Fig. 3H,I), improved liver TG levels in the HFI group could be due to an elevated mitochondrial capacity. In addition, CD36 protein expression was reduced, pointing towards a diminished hepatic fatty acid transport (Fig. 3E–G). HAc feeding also reduced protein levels of CD36 and (in tendency) PPARγ. Expression of PPARγ and genes involved in fatty acid synthesis was reduced by HFI and HAc (Table 2), while gene and protein expression of lipolysis-related enzymes (Atgl and Hsl) were not altered (data not shown). However, Pgc1α and PPARγ gene data are not comparable to the corresponding protein data. This discrepancy might be explained by posttranscriptional regulation of mRNA stability as it is described by Eeckhoute *et al*.^26^ and Aoi *et al*.^27^.

**Table 2:** Inulin and acetate alter hepatic gene expression of enzymes involved in lipogenesis.

	LF	HFC	HFI	HFG	P-value^1^	HF	HAc	HPr	P-value^2^
Srebf1	1.00 ± 0.18	1.03 ± 0.16	0.78 ± 0.09	0.80 ± 0.11	ns	0.80 ± 0.07	0.51 ± 0.10	0.78 ± 0.11	ns
Acly	1.00 ± 0.13	0.85 ± 0.07	0.92 ± 0.12	0.92 ± 0.05	ns	0.68 ± 0.07^a^	0.39 ± 0.04^b^	0.70 ± 0.07^a^	<0.05
Fasn	1.00 ± 0.18	0.75 ± 0.12	0.99 ± 0.14	0.97 ± 0.08	ns	0.63 ± 0.08 ^a^	0.31 ± 0.06^b^	0.64 ± 0.07 ^a^	<0.05
Elovl5	1.00 ± 0.21	1.45 ± 0.20	1.04 ± 0.12	1.68 ± 0.22	ns	1.21 ± 0.12^a^	0.52 ± 0.07^b^	0.90 ± 0.17^a,b^	<0.01
Scd1	1.00 ± 0.16	0.87 ± 0.16	0.82 ± 0.13	1.06 ± 0.25	ns	0.51 ± 0.08^a^	0.15 ± 0.03^b^	0.32 ± 0.07^a,b^	<0.01
Gpam	1.00 ± 0.11	1.14 ± 0.11	1.04 ± 0.09	1.10 ± 0.10	ns	1.06 ± 0.09^a^	0.67 ± 0.12^b^	0.91 ± 0.09^a,b^	<0.05
Mgat1	1.00 ± 0.31	3.14 ± 0.64^a^	0.83 ± 0.15^b^	2.00 ± 0.39^a,b^	<0.05	2.62 ± 0.8^a^	0.30 ± 0.07^b^	0.54 ± 0.09^b^	<0.05
Cidec	1.00 ± 0.23	1.55 ± 0.36^a,b^	0.55 ± 0.05^a^	3.70 ± 0.96^b^	<0.01	1.61 ± 0.57^a^	0.33 ± 0.04^b^	0.66 ± 0.12^a,b^	<0.01

Table shows the summary of relative mRNA expressions of lipid metabolism-related enzymes after 30 weeks of feeding C57BL/6JRj mice with a semi-synthetic low-fat diet (LF) or high-fat diets (HF) supplemented with either 10% dietary fibre (HFC: 10% cellulose; HFI: 3% cellulose + 7% inulin; HFG: 3% cellulose + 7% guar gum) or 5% SCFA with different Ac:Pr ratios, a high acetate (HAc; 10:1 Ac:Pr) or high propionate diet (HPr; 1:2.5 Ac:Pr). Data are mean +/− SEM, n = 8–9, normalized to LF-diet to a value of 1. Statistical tests were performed among ^1^dietary fibre groups (HFC, HFI, HFG) and ^2^SCFA-groups (HF, HAc, HPr).

A reduced hepatic lipogenesis was underpinned by a diminished fatty acid synthase (FASN) activity (Fig. 3J) and *de novo* lipogenesis (DNL)-Index (Fig. 3K) in HAc mice. Despite inducing a similar hepatic phenotype, high Pr administration did not affect any of the investigated mechanisms. However, the brown fat-enriched secreted factor, Nrg4, was shown to attenuate the induction of hepatic de novo lipogenesis^24^. Hence we correlated BAT Nrg4 expression with the hepatic TG content and found an inverse correlation (Supplementary Figure 2B).

### Fermentable dietary fibres change the intestinal microbiota

**Figure 4:**
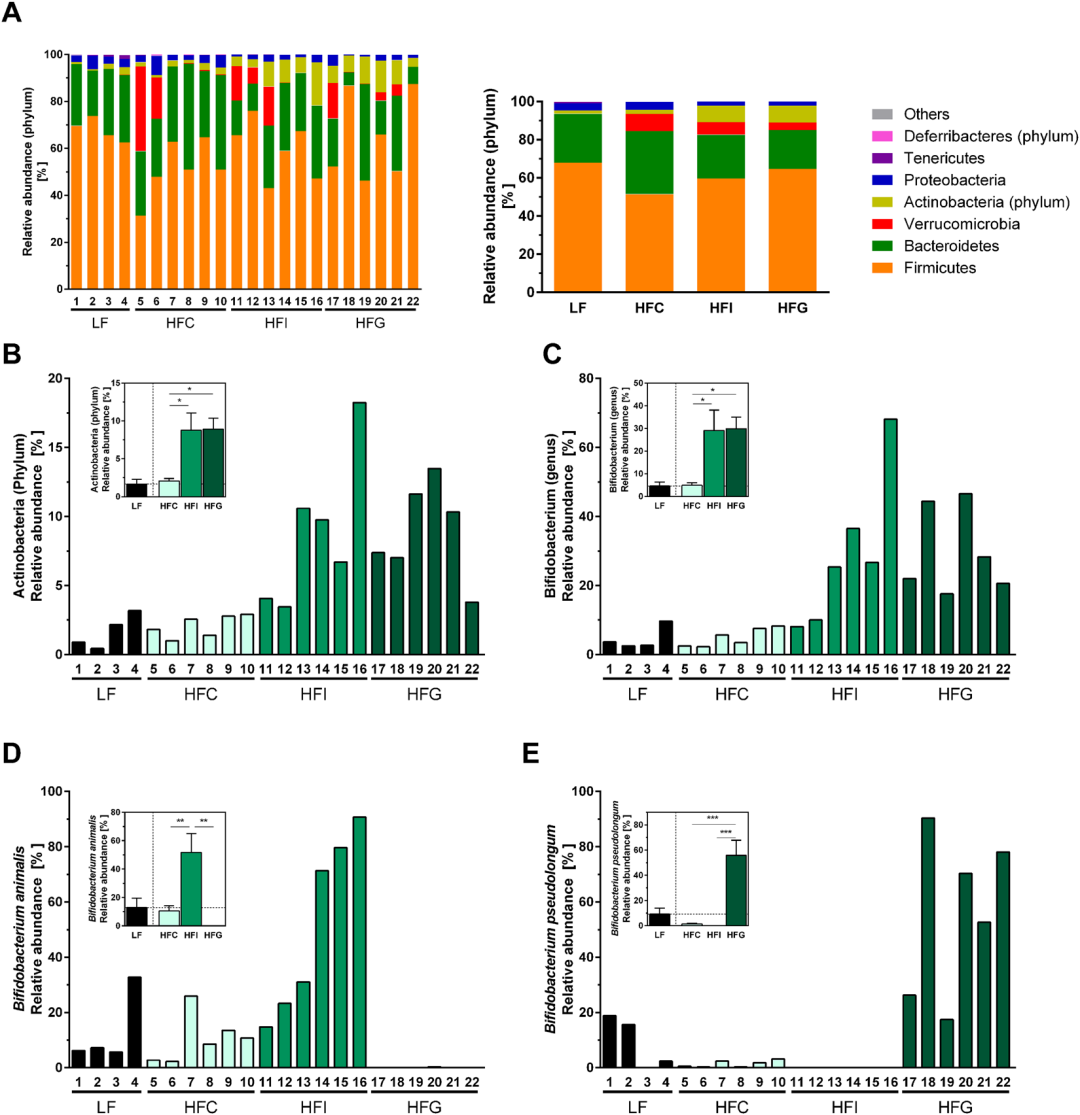
Dietary fibre supplementation modulates the faecal microbiota composition after 30 weeks. Faecal microbiota composition of male C57BL/6JRj mice that were fed a semi-synthetic low-fat diet (LF) or high-fat diets (HF) supplemented with 10% dietary fibre (HFC: 10% cellulose; HFI: 3% cellulose + 7% inulin; HFG: 3% cellulose + 7% guar gum) for 30 weeks. (**A**) Microbial diversity at phylum-level, (**B**) Actinobacteria (phylum), (**C**) Bifidobacterium (genus), (**D**) *Bifidobacterium animalis*, (**E**) *Bifidobacterium pseudolongum*. Data are shown for individual animals (numbered 1–22) and as group mean + SEM (n = 4–7), *p < 0.05, **p < 0.01, ***p < 0.001.

To elucidate if the different effects of long-term (30 weeks) inulin and guar gum feeding on HF diet-induced obesity could be linked to alterations in intestinal microbiota composition, we conducted microbial population analyses in faeces using 16S rRNA gene sequencing. Overall, Firmicutes and Bacteroidetes were the most abundant phyla, with no significant differences among the feeding groups (Fig. 4A). Differences at phylum level were only evident for Actinobacteria (Fig. 4B). The relative abundance of Actinobacteria in both fermentable dietary fibre groups (HFI, HFG) was increased compared to the HFC diet. Inulin and guar gum administration were accompanied by a higher proportion of bifidobacteria (Fig. 4C). This increase in bifidobacteria in the HFI group was mainly due to the proliferation of *Bifidobacterium animalis* (Fig. 4D), whereas that in the HFG group was mainly due to *Bifidobacterium pseudolongum* (Fig. 4E). Overall, dietary SCFA supplementation did not affect faecal microbiota composition on phylum or genus level (Supplementary Figure 3). However, HAc feeding also increased the abundance of *Bifidobacterium animalis*.

## Discussion

Dietary fibre and SCFA are discussed as factors influencing diet-induced obesity and energy homeostasis. Paradoxically, there are data on both, prevention and promotion of obesity development. Studying several types of dietary fibre, we clearly show that fermentability alone is not a decisive criterion in this regard. Furthermore, we highlight the importance of SCFA as metabolic regulators. In particular, the prevention of diet-induced obesity, insulin resistance, and hepatic steatosis by inulin could be completely mimicked by SCFA supplementation in male mice. Despite its reported good fermentability, guar gum supplementation induced none of these beneficial effects. Mechanistically, our data point towards an increased BAT activity and an induction of “beige” adipocytes. Furthermore, we detected changes in the intestinal microbiota and found that each supplement might affect the development of hepatic steatosis through its own specific mechanism.

Based on literature data, it could be assumed that the provision of additional energy by fermentation of dietary fibre is a possible mechanism contributing to the promotion of overweight and obesity^14, 28, 29^. This would imply that all fermentable and soluble dietary fibres promote weight gain during long-term supplementation. Our data do not support this assumption as fermentable inulin protects against diet-induced obesity, while guar gum feeding showed no effect. The protection against diet-induced obesity was even more pronounced in both SCFA-groups, regardless of the Ac:Pr ratio. This suggests that SCFA are mainly responsible for the prevention of HF-induced body weight gain, which is in line with the study of Lin *et al*.^30^. They showed that short-term supplementation of one single SCFA (propionate or butyrate, but not acetate) inhibited weight gain in C57BL/6N mice and connected this effect to a reduced food intake and an increased gut hormone formation. However, den Besten *et al*. recently published that neither Ac nor Pr supplementation affects food intake although they both reduced high-fat diet induced obesity^31^, which is in line with our present study. Here, we depict a non-significant decrease in the daily (data not shown) and 3 day cumulative feed intake which is unlikely to be the main reason for the great body weight differences between the groups. Furthermore, there were no apparent effects on energy intake, energy expenditure and RQ. A reduced secretion of anorexigenic gut hormones, namely glucagon-like peptide-1 (GLP-1) and peptide YY (PYY), is often discussed as one possible reason for a reduced energy intake^32^. Since we did not observe significant effects on the feed intake, we did not determine these gut hormones. However, we cannot exclude that small, non-significant changes in energy intake and expenditure added up in the long run to affect body composition. That dietary fibre does not necessarily affect food intake was also described in a systematic review, which reported that only 39% of all studies demonstrated an effect of dietary fibre intake on appetite regulation^5^.

Here, we confirmed the published beneficial effect of inulin and SCFA on the prevention of HF-induced insulin resistance^20^ and propose that especially Pr plays a crucial role in the underlying mechanism. As previously described^20^, OCFA levels negatively correlated with the insulin area under the curves during OGTT (Supplementary Figure 2C). OCFA are a biomarker for intestinal dietary fibre fermentation and Pr formation^25^, which underlines the particular importance of Pr for the insulin sensitizing effects during the OGTT. The results also indicate that amounts of Pr formed from different types of fermentable dietary fibre vary. While inulin supplementation leads to increased OCFA levels (C15:0 and C17:0), guar gum feeding does not do so. Hence, guar gum has no beneficial effects on postprandial HF-induced insulin resistance, which might be due to the fact that the intestinal fermentation of guar gum is less efficient in propionate production than that of inulin. Owing to methodical problems, we were not able to directly determine caecal SCFA concentrations. However, the lower OCFA levels in the HFG group (compared to HFI) suggest that the formation of propionate is diminished. This possibly also explains that guar gum did not improve HF-induced obesity. Contrary to our results, guar gum treatment was recently reported to decrease various markers of the metabolic syndrome (body/fat weight, triglycerides and HOMA-IR) in a dose-dependent manner^15^. Since caecal SCFA concentrations did not increase in the same dose-dependent manner, the authors hypothesized that the SCFA flux to the metabolic organs rather than the intestinal SCFA concentration is important for the beneficial effects. Although we could not confirm the positive effect of guar gum on HF-induced obesity in the present model, our measurements of hepatic OCFA formation support the importance of circulating SCFA.

Here, we clearly demonstrate that the body weight reduction by inulin and SCFA supplementation is mainly due to a lower fat mass (lean mass was not altered; Table 1). The reduction of adipocyte hypertrophy was more pronounced in sWAT than in eWAT, pointing towards an induction of “beige” adipocyte formation. Indeed, body temperature was increased in HFI and HAc mice about 0.8 °C and 0.6 °C compared to their respective control groups. Interestingly, in these groups body temperature was inversely correlated with the body weight gain during 30 wks of intervention (Supplementary Figure 2D). However, energy expenditure was not affected by these changes in body temperature, which might be due to the different time points of measurement. Whereas energy expenditure was measured during the indirect calorimetry in wk 10, body temperature was determined in wk 29. Hence, energy expenditure was analysed at the beginning of the study, at a time where the effects on adipocyte “browning” and body temperature were maybe not manifested yet.

While gene and protein expression of enzymes involved in fatty acid uptake/transport, synthesis and hydrolysis of triglycerides were not altered in sWAT (data not shown), gene expression of “beige” adipocyte markers (Pgc1α, Ucp1, Cidea) and COX activity were elevated in HFI and HAc mice. BAT activity was also slightly increased in inulin- and HAc-supplemented mice, which is in accordance with recent literature data^11^. Sahuri-Arisoylu *et al*. demonstrated that Ac (applied via i.p. injection of nanoparticles) inhibited lipolysis and induced “browning” in WAT, which in turn led to a reduced obesogenic phenotype^12^. However, despite the fact that the increased body temperature in our study is correlated with a lower body weight, overall evidence for the connection between an increased thermogenic potential and the reduced body weight is limited. Therefore additional studies are needed to further elucidate the effects of dietary fibres and SCFA in more detail.

Interestingly, HPr feeding provided a similar protection against HF-induced obesity as inulin or Ac supplementation but did not increase rectal body temperature, did not induce “browning”, and had no effect on BAT thermogenic capacity. On the other hand, we observed that Pr induced Nrg4 gene expression in BAT *in vivo* and increased secretion of Nrg4 from brown adipocytes *in vitro*. Nrg4 was recently identified as a brown-fat-enriched secreted factor whose expression is highly increased after adrenergic receptor activation^24, 33^. Furthermore, Nrg4 is negatively correlated with body fat mass in humans and Nrg4 deficiency exacerbates diet-induced obesity in a murine model^23, 24^. Adipose tissue-specific overexpression of Nrg4 improved insulin sensitivity and reduced hepatic steatosis^24^. Hence, we correlated Nrg4 expression in BAT with the hepatic TG content and found an inverse correlation. Besides this, also body weight and UCP1 expression were negatively correlated with BAT Nrg4 expression (Supplementary Figure 2E,F). Together, we describe a Pr-dependent induction of Nrg4 in BAT and can link this to an improved hepatic steatosis.

While plasma parameters were not improved after dietary fibre or SCFA supplementation, hepatic triglyceride concentrations were reduced to LF levels in the HFI, HAc and HPr groups. Lee *et al*. described that up-regulation of Pparγ and Mgat1 in hepatocytes are responsible for the induction of hepatic steatosis^34^. In agreement, we found a decreased hepatic gene expression of Pparγ and its target genes (Cd36, Cidec, Mgat1) after HFI and HAc feeding. This was further confirmed by reduced protein levels of CD36, which correlate with lower hepatic triglyceride concentrations in these groups (Supplementary Figure 2G). Hence, our data provide evidence for inulin and acetate being effective in the prevention of hepatic steatosis via a Pparγ-mediated pathway, although phospho-AMPK levels were not altered (data not shown) as previously described^31^. Besides a reduced hepatic fatty acid transport, our data indicate that inulin and Ac supplementation improve liver metabolism through different routes. While inulin increased the mitochondrial activity (indicated by a higher PGC1α protein expression as well as increased enzyme activities of COX and CS), Ac diminished the fatty acid synthesis by reducing FASN activity, DNL-index and gene expression of important lipogenesis enzymes. Given the fact that Liver X receptors (LXRs) are the master regulators of hepatic fatty acid synthesis^35^, the latter is probably due to direct LXR effects or a decreased LXR-dependent up-regulation of Srebf1. Nevertheless, our results are contradictory to another study which suggested that Ac is preferably used for the formation of palmitate leading to the induction of hepatic lipogenesis^6^. Data on the regulation of hepatic β-oxidation by dietary fibre and SCFA are limited^15, 36^. However, an induction of skeletal muscle mitochondrial biogenesis and fatty acid oxidation by viscous dietary fibre was recently described^37^. Moreover, den Besten *et al*. confirmed this finding by showing that supplementation of Ac as well as Pr prevents obesity by stimulating mitochondrial fatty acid oxidation in liver and adipose tissue^31^.

The intestinal microbiota is described as an important factor in the etiology of obesity and its associated disorders^38, 39^. Several studies demonstrate that differences in the Firmicutes to Bacteroides ratios are associated with an obese phenotype. However, published data are not consistent since some studies propose an increased Firmicutes to Bacteroides ratio^40, 41^, while other studies found a reduced ratio^42^ or could even not detect any effect^43^. Using 16S rRNA gene sequencing, we observed no differences in the Firmicutes to Bacteroides ratio among the groups fed different diets. Both inulin and guar gum increased the relative abundance of Actinobacteria which was probably due to an increase in the proportion of bifidobacteria. However, inulin selectively induced *B*. *animalis*, while guar gum promoted the growth of *B*. *pseudolongum*; the abundance of both being negligible in week 0, before the switch to the fibre-rich diets (data not shown). *B*. *animalis* was shown to prevent HF-induced weight gain and glucose intolerance by increasing the intestinal barrier and reducing bacterial translocation^44, 45^. Hence, our data further support the positive effects of *B*. *animalis* and reveal a new mechanism by which inulin could prevent adiposity. *B*. *pseudolongum* was reported to proliferate in response to fructo-oligosaccharide (FOS) supplementation^46, 47^. The authors of this study investigated the prevention of 2,4-dinitrofluorobenzene-induced contact hypersensitivity by FOS and found that *B*. *pseudolongum* is partially responsible for the FOS-induced effects. However, to our knowledge, a role of *B*. *pseudolongum* in obesity development has not yet been reported. Since both, the HFC and HFG diets promote obesity, but only guar gum stimulated the proliferation of *B*. *pseudolongum*, it seems to be unlikely that *B*. *pseudolongum* plays a key role in obesity development. Nevertheless, this needs to be proven by additional experiments using gnotobiotic mice.

**Figure 5:**
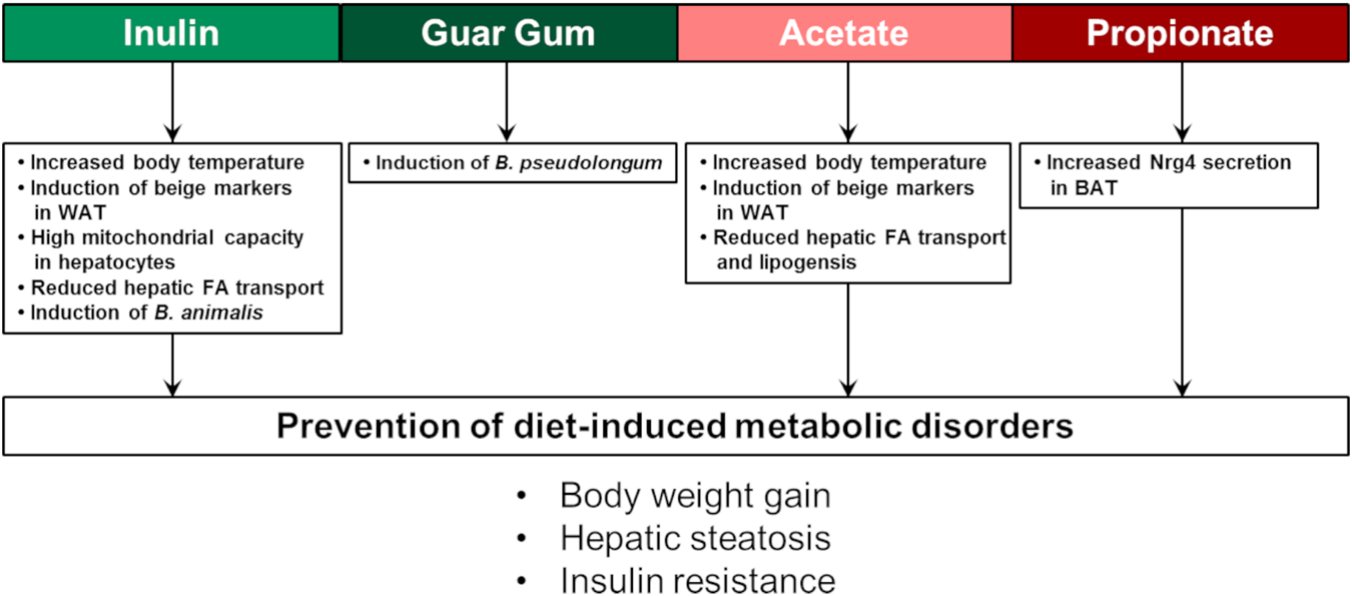
Overview of dietary fibre- and SCFA-mediated metabolic effects to accomplish the prevention of diet-induced metabolic disorders. WAT: White adipose tissue; FA: Fatty acid; Nrg4: Neuregulin 4; BAT: Brown adipose tissue.

Taken together, the present study supports the notion that dietary fibres are quite individual in their capability to reduce HF-induced obesity: While inulin prevented obesity development, guar gum showed no effect at all. Since oral supplementation of SCFA with different Ac:Pr ratios had the same effects as inulin feeding, we suggest that these SCFA are mainly responsible for the positive effects in the HFI group. Nevertheless, each treatment apparently has its characteristic mechanism through which the onset of HF-induced obesity was attenuated (Fig. 5). The present study gives several new insights into the mechanistic action of dietary fibres and their fermentation products. It should be pointed out that our study was not designed to delineate specific effects of acetate and propionate, respectively. For the SCFA supplementation we used different Ac:Pr ratios which should reflect physiological ratios that could possibly be achieved by fermentation of different fibres. Nevertheless, the used Ac:Pr ratios, seem to affect different targets and exert their effects through different molecular mechanisms which act synergistically towards the prevention of diet-induced metabolic disorders. In future studies it would be interesting to focus especially on the role of Nrg4 in the prevention HF-induced obesity by propionate. Furthermore the role of *B*. *animalis* and *B*. *pseudlongum* needs to be further elucidated.

## Materials and Methods

### Animals and experimental setup

All experiments were approved by the ethics committee of the Ministry for Environment, Health, and Consumer Protection of Brandenburg, Germany and the methods were carried out in accordance to approval no. 2347-9-2015. Conventional male C57BL/6JRj mice, 8 wks of age, were purchased from Janvier Labs (Le Genest-Saint-Isle, France). They were group-housed (5–6 animals per cage) in polycarbonate cages in a climate-controlled room (22 ± 2 °C, relative air humidity 55 ± 5%) with a 12-h light:dark cycle. Water and food were applied *ad libitum*. After two wks of adaptation to our animal facility, mice (10 wks old) were switched to semi-synthetic experimental diets (Supplementary Table 1). Animals were either fed a low-fat diet (LF)^48^ or a high-fat diet supplemented with 10% dietary fibre (HFC: 10% cellulose; HFI: 3% cellulose + 7% inulin; HFG: 3% cellulose + 7% guar gum) or 5% SCFA (in the presence of 5% cellulose) with different Ac:Pr ratios, a high acetate (HAc; 10:1 Ac:Pr) or a high propionate diet (HPr; 1:2.5 Ac:Pr). Control mice received the HF diet without SCFA (HF), with the addition of NaCl and CaCO_3_ to adjust dietary sodium and potassium content to those of the SCFA groups. In the fibre-rich diets, a total amount of 10% dietary fibre was chosen based on literature data^14, 49^. In order to reduce the negative side effects (flatulence, diarrhoea) of such a high fermentable fibre intake, we limited inulin or guar gum to 7% and added 3% cellulose to the HFI and HFG diet, while the HFC diet contains 10% cellulose. As previously described^20^, we supplemented the SCFA-containing diets with 5% SCFA. Since it was hypothesised that the Ac:Pr ratio is important for the beneficial effects of dietary fibres^19^, diets were supplemented with two different Ac:Pr ratios (10:1 or 1:2.5) that could possibly be achieved by fermentation of different fibres. After 30 wks of dietary intervention, animals were euthanized with isoflurane and peripheral blood was obtained by cardiac puncture. Tissues were removed, weighed and immediately frozen in liquid nitrogen before they were stored at −80 °C.

### Body composition

Body weight and body composition were determined every four wks and at the end of dietary intervention. Body fat mass was measured with a nuclear magnetic resonance spectrometer EchoMRI™- Analyzer (Echo Medical Systems, Houston, USA). Lean mass was calculated by subtracting fat mass from body weight.

### Calorimetric measurements

Energy contents of diets and feces were determined by bomb calorimetry as previously described^19^. Indirect calorimetry for measurement of energy expenditure was performed in wk 10 using Pheno Master System (TSE Systems GmbH, Bad Homburg, Germany) according to the published procedure^50^.

### Oral glucose tolerance test (OGTT)

In wk 20, after 16 h of fasting (4 pm–8 am), an OGTT was performed as described previously^20^. Briefly, glucose (2 g/kg body weight) was administered orally and blood glucose was measured from tail vein whole blood after 0, 15, 30, 60, 120 and 240 min.

### Plasma and tissue measurements

Plasma measurements were performed in 1:2 diluted samples according to the manufacturer’s instructions. For analysis of circulating non-esterified fatty acids (NEFA) the NEFA C kit (Wako Chemicals GmbH, Neuss, Germany) was used. Plasma cholesterol concentrations were measured using a colorimetric and enzymatic standard method (Cholesterol liquicolor, Human GmbH, Wiesbaden, Germany). Plasma and liver triglycerides (TG) were determined using the TG Determination Kit (Sigma-Aldrich). Liver TG were measured after extraction with 10 mmol/L sodium phosphate buffer (pH 7.4) containing 1 mmol/L EDTA and 1% polyoxyethylene (10) tridecyl ether.

### Histology

Liver and subcutaneous white adipose tissue samples were fixed in 4% paraformaldehyde for 24 h at room temperature, embedded in paraffin, sectioned in 2 µm slices and stained with hematoxylin and eosin (H&E). All images were acquired with an Eclipse E1000 microscope (Nikon Instruments, Amstelveen, Netherlands) and the software Lucia G V.4.82 (Laboratory Imaging Ltd., Prague, Czech Republic). Adipocyte size of H&E stained sWAT was assessed using an Olympus BX 41 color view II microscope and cell^D software. Tissue sections of five animals per group were viewed at 20× magnification. All cells were counted and volumetrically determined. Subsequently, cell size was separated into 30 volumetric subgroups (0–6000 µm^2^, 200 µm^2^) and displayed in percentage.

### Quantitative real-time PCR (qRT-PCR)

RNA extraction, cDNA synthesis and qRT-PCR were performed as previously described^20^. Primer and probe sequences were published in^19^ or summarized in Supplementary Table 2. Gene expression was calculated as ddCT, using the 18S rRNA gene as a reference. Data were expressed relative to the LF-group normalized to a value of 1.

### Western Blot analysis

Protein isolation, immunoblotting and detection were performed as previously described^20, 51^. The following antibodies were used: PGC1α (#sc-13067) and PPARγ (#sc-7196) were purchased from Santa Cruz Biotechnology (Dallas, USA). CD36 (#MAB2519) was obtained from R&D Systems (Minneapolis, USA). As secondary antibodies, anti-rabbit IgG (#7074) and anti-mouse IgG (#7076) (Cell Signaling Technology, Massachusetts, USA) were used. Protein expression was normalized to GAPDH (#AM4300, Thermo Fisher Scientific, Massachusetts, USA).

### Enzyme activity assays

Fatty acid synthase (FASN) activity was determined by a continuous spectrophotometric assay following the conversion of NADPH to NADP by loss of absorbance at 340 nm^52^. Liver tissues (30 mg) were homogenized in 450 µl phosphate-bicarbonate buffer (70 mM KHCO_3_, 85 mM K_2_HPO_4_, 9 mM KH_2_PO_4_, and1 mM dithiothreitol, pH 8.0). After centrifugation (20 min, 23,000 g, 4 °C) FASN activity was determined in the supernatants. In 96-well plates, 20 µl of supernatant, 150 µl of reaction buffer (100 mM potassium phosphate (pH 6.5), 1 mM dithiothreitol, 25 µM acetyl-CoA, and 150 µM NADPH) and 30 µl of 500 µM malonyl-CoA (FASN substrate) were assayed for 10 min at 37 °C. One unit of enzyme activity represents 1 µmol NADPH oxidized per min and protein at 37 °C.

Cytochrome c oxidase (COX) activity and citrate synthase (CS) activity were determined as previously described^53^. For both assays 30 mg of tissue were homogenized in 50 mM Tris, 1 mM EDTA (pH 7.4), and 0.1% Triton X-100. Citrate synthase was determined in 1:6 diluted samples.

### Long-chain fatty acid (LCFA) analysis

Long-chain fatty acid composition was determined in the liver phospholipid fraction by gas chromatography as previously described^19^. The *de novo* lipogenesis (DNL)-index was calculated as the palmitic acid:linoleic acid ratio.

### 16S rRNA gene sequencing

Fresh faecal samples were collected in week 30. Mice were group-housed with 5–6 animals per cage and 16S rRNA analysis was performed from 4–6 animals per group, which originate from at least two different cages. 16S ribosomal RNA (rRNA) gene sequencing was performed by CeMeT GmbH (Tübingen, Germany). Faecal DNA was isolated as described previously^54^ and the V3–V4 region of the 16S rRNA gene was amplified by PCR using 341F/785R primers^55^. Sequencing libraries were prepared with the Nextera XT DNA Library Preparation Kit (Illumina, San Diego, USA). Using the Illumina MiSeq platform, sequencing (min. 10.000 clusters per sample, 2 × 250 bp) was carried out. Reads were merged by using FLASH and subsequently filtered (merged reads <75 bp were removed). The alignment was conducted with MALT using MEGAN LCA parameters (min Support Percent = 0.01 min Score = 400.0 max Expected = 0.0001 min Percent Identity = 0.0 top Percent = 1.0 min Complexity = 0.0 use Identity Filter = true)^56^.

### Primary adipocyte cell culture and Nrg4 measurement

Sca-1 + progenitor cells were isolated from mouse brown adipose tissue by FACS as described before^57^. Fifteen thousand progenitors were plated per well (48 well plate) and pretreated with 1 µM rosiglitazone three days prior to a 48 h adipogenic induction period. After five days of differentiation, fresh medium containing 1 mM of sodium propionate was added and Nrg4 secretion was determined after 4 h incubation by ELISA (SEC174Mu, Cloud-Clone Corp., Houston, USA); as control, cells were incubated with fresh medium without propionate. Nrg4 secretion was normalized to the cellular DNA concentration and relative values were calculated by normalizing the absolute concentrations to the mean of the control. Since literature data of circulating SCFA concentrations are very inconsistent, 1 mM propionate was selected to find a medium path between the possible physiological concentrations and the concentration that was used in other cell culture experiments^31^, bearing in mind that the physiological propionate concentration might be lower.

### Statistical analysis

All data are represented as mean ± SEM. Statistical calculation was performed using GraphPad Prism 6 (GraphPad Software, La Jolla, USA). Comparisons were performed between dietary fibre groups (HFC, HFI, HFG) and SCFA-groups (HF, HAc, HPr). Normal distribution and homogeneity of variances were tested using the Kolmogorov-Smirnov test. Normally distributed data were analysed by using an ordinary one-way analysis of variance (ANOVA) with Bonferroni’s posttest. Non-normally distributed data were analysed using nonparametric Kruskal-Wallis test. Differences with P < 0.05 were considered statistically significant.

### Data Availability

The datasets generated during and/or analysed during the current study are available from the corresponding author on reasonable request.

## Electronic supplementary material

Supplementary data
