# Supplementary material for: Short-chain fatty acids and inulin, but not guar gum, prevent diet-induced obesity and insulin resistance through differential mechanisms in mice
Source: Sci Rep. 2017 Jul 21;7:6109. doi: 10.1038/s41598-017-06447-x (PMC5522422; doi:10.1038/s41598-017-06447-x)
Supplement: Supplementary file 1 — Supplementary data [file 41598_2017_6447_MOESM1_ESM.doc]

**Short-chain fatty acids and inulin, but not guar gum, prevent diet-induced obesity and insulin resistance through differential mechanisms in mice**

Karolin Weitkunat^a^, Christin Stuhlmann^a^, Anna Postel^a^, Sandra Rumberger^a,b^, Maria Fankhänel^a^, Anni Woting^b^, Klaus Jürgen Petzke^a^, Sabrina Gohlke^b^, Tim J. Schulz^b^, Michael Blaut^c^, Susanne Klaus^a^, Sara Schumann^a^

**Supplementary table 1:** Composition of semi-synthetic experimental low-fat diet (LF) or high-fat diets (HF) supplemented with either 10% dietary fibre (HFC: 10% cellulose; HFI: 3% cellulose + 7% inulin; HFG: 3% cellulose + 7% guar gum) or 5% SCFA with different Ac:Pr ratios, a high acetate (HAc; 10:1 Ac:Pr) or high propionate diet (HPr; 1:2.5 Ac:Pr).

| Component | LF | HFC | HFI | HFG | HF | HAc | HPr |
| --- | --- | --- | --- | --- | --- | --- | --- |
|  | (g/kg) | (g/kg) | (g/kg) | (g/kg) | (g/kg) | (g/kg) | (g/kg) |
| Casein | 220 | 267 | 267 | 267 | 267 | 267 | 267 |
| Wheat starch | 386,5 | 122,5 | 122,5 | 122,5 | 127,1 | 101,0 | 99,9 |
| Maltodextrin | 100 | 100 | 100 | 100 | 100 | 100 | 100 |
| Dextrose | 50 | 50 | 50 | 50 | 50 | 50 | 50 |
| Sucrose | 100 | 100 | 100 | 100 | 100 | 100 | 100 |
| Coconut oil | 7,7 | 37,8 | 37,8 | 37,8 | 37,8 | 37,8 | 37,8 |
| Sunflower oil | 30,1 | 147,0 | 147,0 | 147,0 | 147,0 | 147,0 | 147,0 |
| Linseed oil | 5,2 | 25,2 | 25,2 | 25,2 | 25,2 | 25,2 | 25,2 |
| Cellulose | 50 | 100 | 30 | 30 | 50 | 50 | 50 |
| Inulin^a)^ | - | - | 70 | - | - | - | - |
| Guar gum^b)^ | - | - | - | 70 | - | - | - |
| Acetate | - | - | - | - | - | 65,6 | 20,6 |
| Sodium acetate^c)^ | - | - | - | - | - | 32,8 | 10,3 |
| Calcium acetate monohydrate^c)^ | - | - | - | - | - | 32,8 | 10,3 |
| Propionate | - | - | - | - | - | 5,9 | 46,3 |
| Sodium propionate^b)^ | - | - | - | - | - | 2,9 | 23,2 |
| Calcium propionate^b)^ | - | - | - | - | - | 3,0 | 23,1 |
| NaCl^c)^ | - | - | - | - | 25,2 | - | 3,7 |
| CaCO_3_^c)^ | - | - | - | - | 20,3 | - | 2,0 |
| Mineral Mix | 35 | 35 | 35 | 35 | 35 | 35 | 35 |
| Vitamin Mix | 10 | 10 | 10 | 10 | 10 | 10 | 10 |
| Choline tartrate | 2,5 | 2,5 | 2,5 | 2,5 | 2,5 | 2,5 | 2,5 |
| L-Cysteine | 3 | 3 | 3 | 3 | 3 | 3 | 3 |

a) Fibruline DS2, Georg Breuer GmbH, Königstein, Germany, b) Sigma-Aldrich, Steinheim, Germany, c) Carl Roth GmbH + Co. KG, Karlsruhe, Germany. Diets were mixed and pelletized by SSniff GmbH, Soest, Germany.

**Supplementary table 2:** Oligonucleotides used in this study to measure mRNA levels.

| **Primer name** |  | **Primer sequence (5´🡪3´)** |
| --- | --- | --- |
| 18S rRNA | f | ACC ACA TCC AAG GAA GGC AG |
|  | r | TTT TCG TCA CTA CCT CCC C |
|  | p | 6-FAM-AGG CGC GCA AAT TAC CCA CTC CC-TAMRA |
| Pparγ | f | TGC CAA AAA TAT CCC TGG TT |
|  | r | GGC GGT CTC CAC TGA GAA TA |
| Mgat1 | f | GGAGGTGGCAATGTCTCAAT |
|  | r | TGGGTCAAGGCCATCTTAAC |
| Cidec | f | GTA TTG CCA GGA GGC TGG G |
|  | r | GGG TAC AGG AGG CTG AGA GA |
| Atgl | f | AAC ACC AGC ATC CAG TTC AA |
|  | r | GGT TCA GTA GGC CAT TCC TC |
|  | p | 6-FAM-CGA AGG CTC TCT TCC CGC CA-TAMRA |
| Hsl | f | GCT TGG TTC AAC TGG AGA GC |
|  | r | TGC CTC TGT CCC TGA ATA GG |
|  | p | 6-FAM-CTG CTG CCC GAA GGG ACA CA-TAMRA |
| Pgc1α | f | CTA CAG ACA CCG CAC ACA CC |
|  | r | GCG CTC TTC AAT TGC TTT CT |
| Ucp1 | f | AGT ACC CAA GCG TAC CAA GC |
|  | r | AGA AGC CAC AAA CCC TTT GA |
|  | p | 6-FAM-AAG GCC GTC GGT CCT TCC TTG-TAMRA |
| Cidea | f | TGC TCT TCT GTA TCG CCC AGT |
|  | r | GCC GTG TTA AGG AAT CTG CTG |
| Nrg4 | f | ACA CTT GTG AAA CGC TGC AT |
|  | r | GCT CGT GAT CTG TTG GCA TC |
| Tfam | f | AAG ACC TCG TTC AGC ATA TAA CAT T |
|  | r | TTT TCC AAG CCT CAT TTA CAA GC |
| Leptin | f | TTC ACA CAC GCA GTC GGT AT |
|  | r | TCA TTG GCT ATC TGC AGC AC |
|  | p | 6-FAM-CCT CAC CAG CCT GCC TTC CC-TAMRA |
| Vegfα | f | CTG TAA CGA TGA AGC CCT GGA G |
|  | r | TGG TGA GGT TTG ATC CGC AT |

**
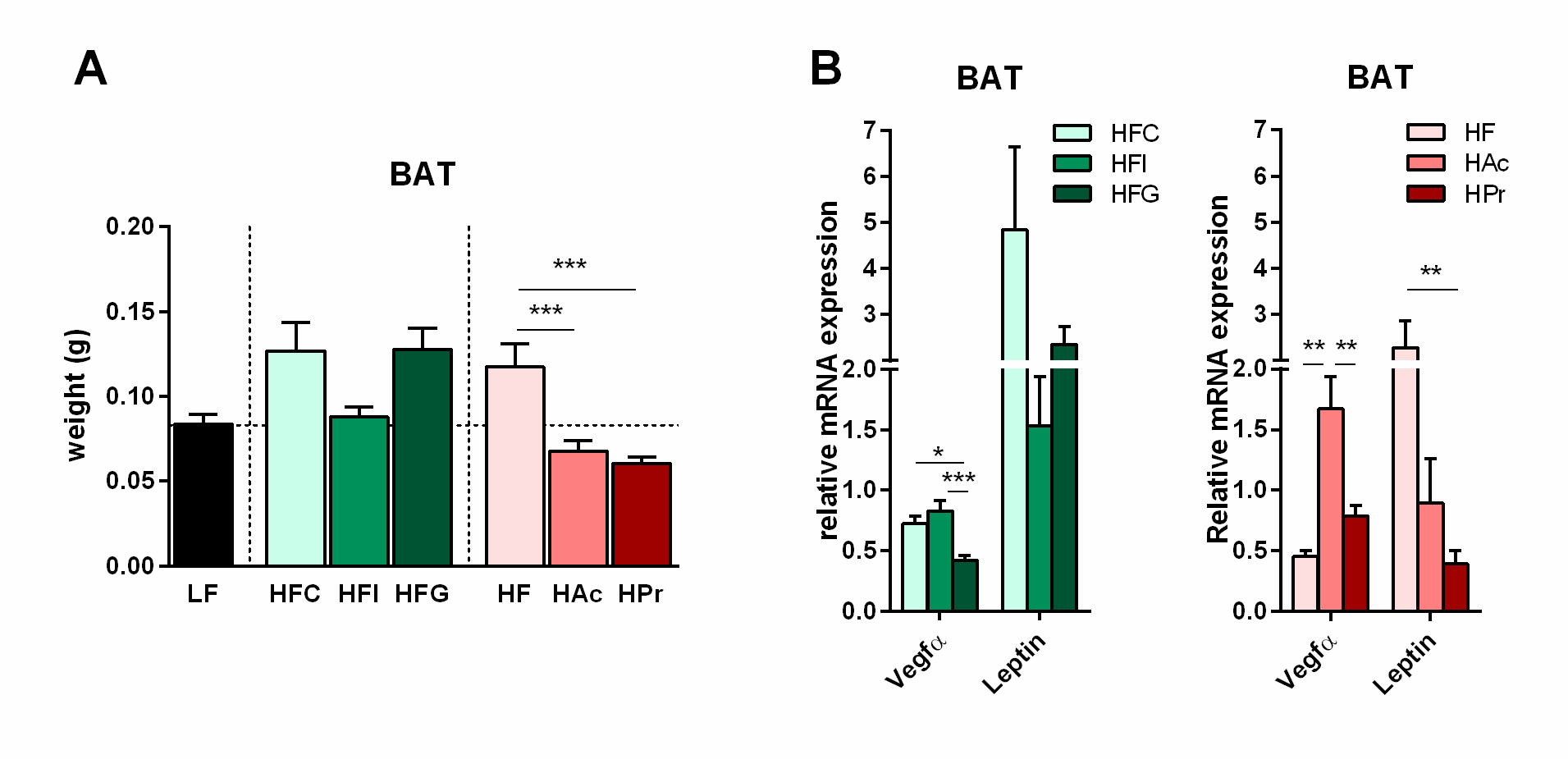
Supplementary figure 1: Ectopic fat accumulation in brown adipose tissue is prevented by inulin and SCFA.** C57BL/6JRj mice were fed a semi-synthetic low-fat diet (LF) or high-fat diets (HF) supplemented with either 10% dietary fibre (HFC: 10% cellulose; HFI: 3% cellulose + 7% inulin; HFG: 3% cellulose + 7% guar gum; depicted in green hues) or 5% SCFA (depicted in red hues) with different Ac:Pr ratios, a high acetate (HAc; 10:1 Ac:Pr) or high propionate diet (HPr; 1:2.5 Ac:Pr). (A) Brown adipose tissue (BAT) weight and (B) corresponding mRNA expression of BAT whitening markers after 30 weeks of intervention, n=9-11. Data are mean +/- SEM, *P<0.05; **P<0.01.

**Supplementary figure 2: Pearson correlation of various parameters.** Correlation analysis of C57BL/6JRj mice that were fed a semi-synthetic low-fat diet (LF) or high-fat diets (HF) supplemented with either 10% dietary fibre (HFC: 10% cellulose; HFI: 3% cellulose + 7% inulin; HFG: 3% cellulose + 7% guar gum) or 5% SCFA with different Ac:Pr ratios, a high acetate (HAc; 10:1 Ac:Pr) or high propionate diet (HPr; 1:2.5 Ac:Pr) for 30 weeks. (A) relative mRNA expression of neuregulin 4 (Nrg4) in BAT and OCFA formation in liver, (B) relative mRNA expression of Nrg4 in BAT and hepatic triglyceride (TG) concentration, (C) OCFA formation in liver and incremental area under the curve (iAUC) of insulin secretion during the oral glucose tolerance test (OGTT), (D) rectal body temperature in week 29 and Δ body weight gain (wk 30 - wk 0), (E) relative mRNA expression of Nrg4 in BAT and final body weight in wk 30, (F) relative mRNA expression of Nrg4 and Ucp1 in BAT (G) relative protein expression of CD36 in liver and hepatic TG concentration.

**Supplementary figure 3: Faecal microbiota composition in SCFA supplemented groups after 30 weeks of intervention.** Microbial composition of C57BL/6JRj mice that were fed a semi-synthetic low-fat diet (LF) or high-fat diets (HF) supplemented with 5% SCFA of different Ac:Pr ratios, a high acetate (HAc; 10:1 Ac:Pr) or high propionate diet (HPr; 1:2.5 Ac:Pr) for 30 weeks. (A) Microbial diversity at phylum-level, (B) Actinobacteria (phylum), (C) Bifidobacterium (genus), (D) *Bifidobacterium animalis*, (E) *Bifidobacterium pseudolongum*. Data are illustrated for each animal separately or as mean + SEM (n=4-7), *p < 0.05, **p < 0.01, ***p < 0.001.
